# Diabetes Data Management System to Improve Glycemic Control in People With Type 1 Diabetes: Prospective Cohort Study

**DOI:** 10.2196/mhealth.8532

**Published:** 2017-11-21

**Authors:** Concetta Irace, Matthias Axel Schweitzer, Cesare Tripolino, Faustina Barbara Scavelli, Agostino Gnasso

**Affiliations:** ^1^ Metabolic Diseases Unit Department of Health Science University of Catanzaro Catanzaro Italy; ^2^ Roche Diagnostics GmbH Mannheim Germany; ^3^ Metabolic Diseases Unit Department of Clinical and Experimental Medicine University of Catanzaro Catanzaro Italy

**Keywords:** diabetes mellitus, blood glucose self-monitoring, smartphone, internet

## Abstract

**Background:**

Smartphone and Web technology can improve the health care process, especially in chronic diseases.

**Objective:**

The aim of this study was to investigate whether the use of blood glucose (BG) data management system, which enables connection to smartphones, the Web, the cloud, and downloading, can improve glycemic control in subjects with type 1 diabetes mellitus (T1DM).

**Methods:**

This study was a prospective, single-arm, cohort feasibility study with 6 months of duration. T1DM subjects enrolled had experience in self-monitoring blood glucose, but were download data naïve. Fasting BG and glycated hemoglobin (HbA_1c_) were collected at the enrollment and at follow-up. Subjects were divided into Downloader (DL) and No-downloader (NDL).

**Results:**

A total of 63 subjects were analyzed, of which 30 were classified as DL and 33 as NDL. At the end of the study, DL had significantly lower HbA_1c_, mean daily glucose, standard deviation, percentage of BG values above target, and pre- and postprandial (lunch and dinner) values compared with NDL (all *P*<.05). The percentage of BG values within treatment target was significantly higher in DL compared with NDL (47% [SD 9] vs 37% [SD 13]; *P*=.001).

**Conclusions:**

The findings suggest that, in T1DM, downloading of BG from data management system, which enables connection to smartphones, the Web, and the cloud, might be a valuable contributor to improved glycemic control.

## Introduction

### Background

Optimizing insulin therapy and achieving good metabolic control is still a challenge in the management of type 1 diabetes mellitus (T1DM). Indeed, subjects with T1DM experience higher glycemic variability than those with type 2 diabetes mellitus (T2DM), and this variability is associated with higher risk of hypoglycemia and worse metabolic control [1]. Self-monitoring blood glucose (SMBG) provides real-time information to patients, allowing adjustment of therapy and also prevention of hypoglycemia in everyday life and during specific conditions such as physical activity, stress, and illness. It also allows sufficient interaction between patients and the health care team to analyze blood glucose (BG) data and to evaluate glycemic trends, glycemic variability, and the risk of hypoglycemia and hyperglycemia [2-4]. So far, many studies have demonstrated the efficacy of SMBG in improving decision making, obtaining better glycemic control, and facilitating a more timely and aggressive change of diabetes therapy, as well as in starting insulin therapy both in type 1 and type 2 diabetes [5-7]. These studies have stressed the need of availability of sufficient BG data, to involve caregivers and patients in the management of the disease, and to share information to achieve a good and stable metabolic control. The idea arising from these trials is that health care providers (HCPs), caregivers, and patients should be in close collaboration for optimal diabetes therapy and outcome. An overall prerequisite is the use of BG data or information. BG information can be gathered through new technologies such as the Internet-enabled BG meter connected to computer systems, mobile phones, and the Web.

The management of diabetes requires some basic steps and rules such as knowing target BG values, gathering glucose data, interpreting glycemic patterns, and taking therapeutic action [8,9].

However, once BG data have been collected by the patient, downloading of the data, using them, and sharing them with HCPs is still limited and sometimes overcomplicated. In clinical practice, BG data in many cases are still shown during the scheduled office visits only or maybe forwarded in advance by fax or mail or via social media. New technologies such as smartphone apps and Web-enabled systems are more and more commonly used to connect all people involved in the management and monitoring of diabetes therapy. Recently, a new connected, Web- and cloud-based system, the Accu-Chek Connect diabetes management system (DMS), has been developed, with an aim to improve collection and management of BG data [10]. The system consists of 3 elements, the BG meter with Bluetooth low energy connectivity, smartphone apps, and a respective Web portal, all of which are wirelessly connected. Patients and caregivers check and tag BG with the meter and download the data into the app and the portal. A dedicated software provides analysis of the glucose data and generates different reports to visualize the information and pattern. Once glucose data are downloaded, HCPs can access the data in the cloud after logging onto the system with their personal account. Analyzed BG data, glycemic trends, hypo- and hyperglycemic events, glucose variability, and mean BG values can be detected. Ease of use and time efficiency of the Accu-Chek Connect DMS have been demonstrated by HCPs, patients with diabetes, and caregivers to obtain information, interpret data, and make therapy decisions [10]. HCPs assessed the system easily and quickly to identify glycemic pattern and to take therapeutic decisions. Identification and therapy decision making was also done in a shorter time compared with traditional BG logbook approaches.

### Objective

On the basis of this knowledge and those findings, we have designed a prospective, single-arm, cohort feasibility study in subjects with T1DM using a connected BG data management system and put the primary objective and the focus of the analysis of this study on the relationship between BG download or no-download and the impact of frequency of BG download on glycemic control and therapy success. The hypothesis of the study was that BG downloading has a positive effect on diabetes therapy success and glycemic control, which would go along with findings from previous clinical studies but would also be true and maybe even advanced using a connected BG data management system such as the Accu-Chek Connect DMS.

## Methods

### Subjects and Study Design

A prospective, single-arm, cohort feasibility study was conducted including consecutive adult subjects with T1DM, who visited our hospital from January to June 2015 and met the inclusion and exclusion criteria. Inclusion criteria were as follows: age ≥18 years, diagnosis of T1DM, glycated hemoglobin (HbA_1c_) <10%, recent admission to our clinic (≤6 months), ability to perform SMBG and carbohydrates counting or alternative way to adjust bolus insulin before meals, current use of BG meters connecting with the Accu-Chek Connect Smartphone app and the Accu-Chek Connect Web portal, and knowledge on how to download BG data into the system, but so far download naïve.

Exclusion criteria were as follows: current use of continuous glucose monitoring; pregnancy; alcohol consumption exceeding 20 grams per day; clinical conditions requiring intensive insulin treatment such as infection, surgery, and acute vascular event; concomitant corticosteroid treatment; and hyper- or hypothyroidism.

The protocol of the study was submitted to the local ethical committee, and the research was conducted according to local legal requirements and good clinical practice. Eligible patients were included in the study after signing informed consent. Data were collected at baseline and after 6 months (follow-up visit at the end of the study).

Participants were asked to perform self-monitoring following previous habits and download data into the system at least once during the 6-month period. Patients were not encouraged to examine and interpret reports following data downloading. Physicians analyzed downloaded data and suggested therapeutic changes when appropriate and according to clinical guidelines.

Office visits were scheduled as suggested by the conventional standard of diabetes care. If necessary, adjustment of ongoing therapy was communicated to the patient after reviewing the download of SMBG.

At 6-month follow-up visit (end of the study), primary analysis was made from two groups: those who downloaded the data from the BG meter into the system (Downloader, DL) at least one time from enrollment to the follow-up visit and those who did not download the data (No-downloader, NDL). Secondary analyses were made specifically on the impact of frequency of downloading on glycemic control and the impact of downloading in subjects on insulin pump therapy (continuous subcutaneous insulin injection, CSII) as a special subgroup.

On the basis of the educational level, subjects were defined as “student,” “graduated,” and “not-graduated.”

### Accu-Chek Connect DMS and Glycemic Outcomes

Accu-Check Connect DMS (Roche, Indianapolis, IN) consists of 3 elements: the BG meter, the smartphone app, and the Web portal. The meter allows measurement of BG values, storing the values in the meter and connecting and transmitting wirelessly the values to the smartphone app. BG data can be sent from the smartphone app to the Web portal or can also directly be downloaded from the BG meter into the portal. The smartphone app can generate messages and automatic reports, for example, a 3-day glycemic trend derived from Structured SMBG. SMBG data, once downloaded, are stored in the cloud and are accessible for the HCP after signing in to the Web portal system. The Web portal is also able to generate automatic messages reporting that the patient file has been updated. The underlying software outputs different analyses and BG data visualization, illustrating glycemic pattern and variables, as well as a traditional logbook design.

### Biochemical Variables

Fasting blood glucose (FBG) and HbA_1c_ were measured as recommended by the national guidelines. FBG was measured by the glucose-hexokinase method (Roche, Base, Switzerland); HbA_1c_ was measured with a high-performance liquid chromatographer standardized and aligned to the United Kingdom Prospective Diabetes Study (UKPDS) and the Diabetes Chronic Complications Trial (DCCT) (Menarini, Florence, Italy). For this study, we collected data at baseline and after 6 months.

### Statistical Analyses

Statistical analyses were performed using PASW 18.0 for Windows (SPSS, Quarry Bay, HK). Variables not normally distributed were as follows: total daily insulin, percentage of values below the target and within the target, absolute and percentage difference of HbA_1c_ between follow-up and baseline visit, and mean postprandial (lunch and dinner) glucose. A 2-step rank transformation was performed to normalize these variables before applying parametric tests. The *t* test for unpaired data was used to compare means between two groups, and the chi-square test was used to compare percentages. The *t* test for paired data was used to compare variables measured at baseline and follow-up visit within each group.

## Results

A total of 63 subjects were enrolled in the study, 44% (28/63) males, aged between 18 and 60 years. Moreover, 52% of participants (33/63) were treated with CSII with insulin pumps, and 48% (30/63) with multiple daily insulin injection. The prevalence of students was 41% (26/63), graduated 16% (10/63), and not-graduated 43% (27/63) (graduated and not-graduated are reported as nonstudent). Among nonstudent participants 31% (11/37), were unemployed, 40% (15/37) employed, and 29% (11/37) independent professional.

On the basis of the download of BG data, subjects were divided into two groups: DL (48%, 30/63) and NDL (52%, 33/63). Among DL, 63% (19/30) subjects downloaded the BG data one time from the baseline to follow-up visit, 34% (10/30) downloaded 2 times, and 3% (1/30) downloaded 3 times. All data downloaded into the system were reviewed by the physicians, and if necessary, therapy was changed accordingly following the current practice.

Characteristics of subjects included in the study at the time of the enrollment, grouped as DL and NDL, are reported in Table 1.

Age, disease duration, and prevalence of male sex were comparable between the groups. The percentage of subjects on CSII therapy was higher in DL compared with NDL, even if the difference was not statistically significant. The prevalence of unemployed, employed, and independent professional in the two groups, respectively, was as follows: DL 19% (6/30), 62% (18/30), and 19% (6/30); NDL 42% (14/33), 37% (12/33), and 21% (7/33), (*P*=.04).

Table 2 shows biochemical and clinical characteristics of DL and NDL collected at baseline and at the follow-up visit.

At baseline, no statistically significant difference between DL and NDL was observed with regard to glycemic control and BG data. At follow-up, DL had significantly lower FBG and HbA_1c_ compared with NDL. HbA_1c_ significantly decreased in DL at follow-up but remained unchanged in NDL.

**Table 1:** Characteristics of subjects enrolled in the study and grouped as No-downloader (NDL) and Downloader (DL).

Variable	No-downloader (N=33)	Downloader (N=30)
Males, n (%)	14 (42)	14 (46)
Age in years, mean (SD)	29.4 (13.4)	28.8 (12.2)
Disease duration in years, mean (SD)	14.3 (8.4)	17.5 (10.3)
CSII^a^, n (%)	15 (45)	18 (60)
Student, n (%)	14 (43)	12 (40)
Graduated, n (%)	7 (21)	3 (10)
Not-graduated, n (%)	12 (36)	50

^a^CSII: continuous subcutaneous insulin injection.

**Table 2:** Fasting blood glucose, glycated hemoglobin, body weight, and total daily insulin at baseline and follow-up visit in subjects enrolled in the study and divided as No-downloader and Downloader.

Variables	Baseline		Follow-up	
	No-downloader (N=33) mean (SD)	Downloader (N=30) mean (SD)	No-downloader (N=33) mean (SD)	Downloader (N=30) mean (SD)
FBG^a^, mg/dL	160 (58)	150 (43)	166 (51)	143 (36)^b^
HbA_1c_^c^, %	7.85 (0.62)	7.51 (0.71)	7.95 (0.74)	7.38 (0.66)^b,d^
Body weight, kg	68.9 (13.6)	69.2 (14.7)	69.4 (13.9)	70.6 (14.4)
TDI^e^, unit/day	46.3 (18.3)	47.3 (16.8)	41.9 (15.0)	40.2 (14.8)
Unit per kg body weight	0.67	0.68	0.60	0.57

^a^FBG: fasting blood glucose.

^b^*P*=.003 versus NDL (unpaired *t* test).

^c^HbA_1c_: glycated hemoglobin.

^d^*P*=.045 versus baseline.

^e^TDI: total daily insulin.

**Figure 1:**
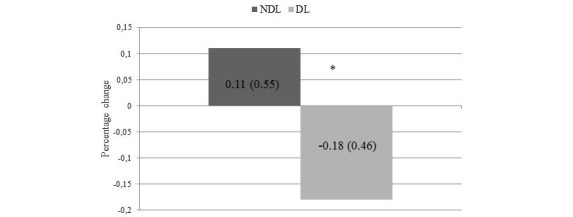
Absolute change in glycated hemoglobin (HbA_1c_) between baseline and follow-up visit in No-downloader (NDL) and Downloader (DL). Values are expressed as mean (SD). *P=.03.

Figure 1 shows the absolute change of HbA_1c_ between baseline and follow-up visit in DL and NDL.

**Figure 2:**
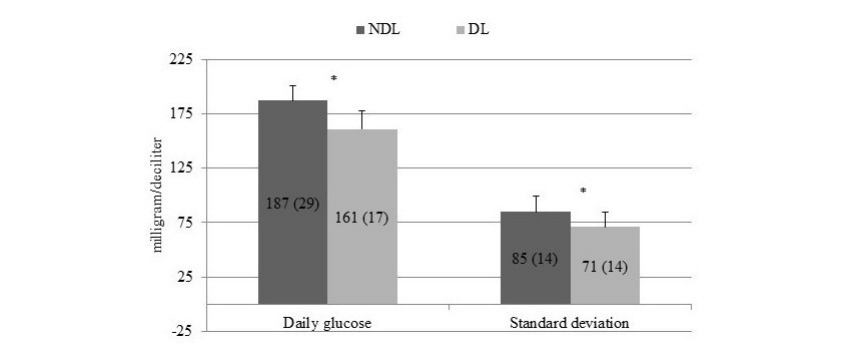
Mean (SD) daily glucose and mean standard deviation of No-downloader (NDL) and Downloader (DL) at follow-up visit. *P=.001.

**Figure 3:**
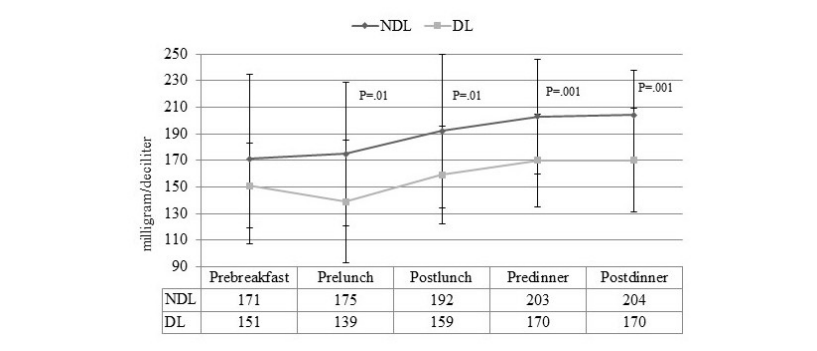
Self-monitoring blood glucose of No-downloader (NDL) and Downloader (DL) at follow-up visit.

**Figure 4:**
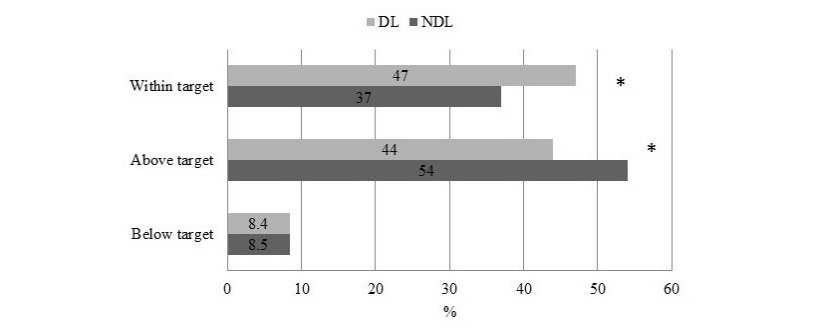
Percentage of values within, above, and below target of No-downloader (NDL) and Downloader (DL) at follow-up visit. *P=.01.

Glycemic variables generated by the BG data management system at follow-up visit as mean of the last 4 weeks are displayed in Figures 2-4.

Figure 2 shows the mean daily glucose and standard deviation, and both were significantly lower in DL compared with NDL. Premeal and postmeal (lunch and dinner) glucose were significantly different between the two groups, whereas prebreakfast glucose was comparable (Figure 3). Figure 4 illustrates the percentage of values within, above, and below target.

At follow-up visit, the percentage of values within the target was significantly higher, and the percentage of values above the target was significantly lower in DL. The prevalence of values below the target was comparable between the two groups. The mean number and (SD) of BG testing per day was comparable between DL and NDL, 4.2 (1.5) versus 3.7 (1.4), *P*=.21.

The same analyses, comparing DL versus NDL, were also performed in patients on CSII therapy, of which 17 were DL and 13 NDL. Mean HbA_1c_ and (SD) at baseline and follow-up visit were 7.6 (0.8) versus 7.4 (0.8) % in DL, and 8.2 (0.4) versus 8.1 (0.6) % in NDL, respectively. Mean absolute difference was −0.26 (0.55) in DL and −0.10 (0.51) in NDL (*P*=.39).

To verify whether the frequency of downloads performed during the study period would influence HbA_1c_ and therapy success, patients were divided into two groups: those who downloaded BG one time (19/30) and those who downloaded ≥2 times (11/30). Mean (SD) HbA_1c_ at baseline and follow-up were, respectively, 7.3 (0.6) versus 7.1 (0.3) % in subjects who downloaded one time, and 7.8 (0.7) versus 7.7 (0.5) % in subjects who downloaded ≥2 times. Mean absolute difference was −0.26 (0.53) in those who downloaded one time and −0.10 (0.29) in those who downloaded ≥2 times (*P*=.22).

## Discussion

### Principal Findings

This study has demonstrated that a new BG data management system, the Accu-Chek Connect DMS, designed to collect, analyze, download, and share BG data, may offer benefits to improve the management of the disease. Indeed, the main finding of this prospective cohort study is the significant difference in HbA_1c_ from baseline to follow-up visit between patients who used the BG information better, DL compared with NDL, with an overall difference of approximately 0.3%. The change might seem modest but is worthy of consideration.

Other BG parameters measured or analyzed in this study, that is, mean daily glucose, standard deviation, and other BG values showed similar advances in DL compared with NDL, all consistent with the difference in HbA_1c_.

T1DM and T2DM require optimal glycemic control to prevent acute and chronic complications [11,12]. Over the years, technology has supported more and more diabetes monitoring and management. BG meters and software enabling downloading of BG data are tools potentially helping to overcome the limits and drawbacks of manual BG recording to make the data more intelligible and usable [13-15]. Clinical studies with SMBG performed in a structured manner (structured testing) confirmed improvement of glycemic control and therapy success. These findings were based on better quality of BG information, better use of BG information available, and BG information sharing with HCP. Therapy change and decision making were more frequent and aggressive when structured testing was approached. Today’s evidence supports that diabetes therapy success is largely dependent on the amount of glucose information available (frequency), the quality of information (eg, structured testing and analyses), and the use of BG data (download and data sharing) in daily diabetes management.

New technology might support better analyses of BG data and better downloading, availability, and use of BG data, thereby improving decision-making and glycemic outcomes [16].

Today, there is much debate about which BG parameters generated by BG data management systems can be more useful for the assessment of glycemic control and the management of the disease. In this regard, some considerations may be useful. Mean value might not be representative of glycemic variability because BG data are generally not normally distributed and the mean value is affected by the number of observations, single outlier, or aberrant values [17,18]. However, based on our results and previous studies, we suggest that BG parameters generated by the Accu-Chek Connect DMS from BG data might assist medical decision making in addition to traditional BG analysis and HbA_1c_.

Furthermore, another reason that might contribute to making the BG data management systems able to positively impact glycemic control is the ability of those systems to store and facilitate sharing of the data. Once the patient with diabetes has downloaded the data into the cloud, an automatic message is generated, and in turn, the HCP is able to update subject files, further analyze BG, and, if necessary, contact the patient and give suggestion and advice about therapy or even adopt or escalate medication. We have not reported the results on how and when the therapy of subjects has been modified because that was not the aim of our study. However, sharing information through the BG data management system might promote the contact between patients and HCPs and allow use of glucose data for therapy advice or optimization whenever it is needed. The recent paper by Chow et al has reported that the frequency of online communication with HCP, along with an adequate number of tests per day (twice or more), is associated with a lower HbA_1c_ in T2DM patients on oral medication. In other words, the number of tests per day matters, but if glycemic values are frequently communicated via Internet to the physician, the efficacy of the numbers in terms of HbA_1c_ reduction is greater [19].

We have designed our study as a prospective study and included subjects with T1DM who benefit from close relationship with their HCPs, continuous support in insulin dose adjustment, high level of disease knowledge, and motivation of constant self-management. In this specific scenario, the remote data management might contribute and support those needs and enable close collaboration between patients with diabetes and HCPs. The ability of collecting, downloading, and sharing BG data can improve timely availability and the use of BG data and enhance the achievement of a better glycemic control. The comparison of DL versus NDL stands for availability and use of BG data, and improvement or difference in HbA_1c_ as found in the study was largely expected. The improvement in glycemic control was a consistent finding in the overall study population and in the CSII subgroup even if the result lacked significance in that subpopulation, probably because of the low number of subjects. In addition to the download of the data, the improvement of HbA_1c_ was probably because of the review and interpretation of the data. Indeed, in our study, all data were reviewed by a physician and, if necessary, the therapy was changed.

We did not find any difference between DL and NDL in the percentage of hypoglycemic events, defined as capillary glucose lower than 70 mg/dL. Hypoglycemia is a very common event in T1DM and the pathogenesis is very complex. It has been estimated that each individual experiences about 2 episodes of symptomatic hypoglycemia per week in real life [20]. As far as our results are concerned, we might argue that a more frequent downloading, or an active interpretation of the results by the patient, should offer the opportunity to significantly decrease the number of hypoglycemic events. Similarly, we did not find any significant difference of HbA_1c_ between subjects who downloaded once and those who downloaded more than once during the observation period. Due to the small sample size and the fact that patients essentially downloaded only 1 or 2 times in the 6 months, we could not assess whether more frequent downloading or reporting would have impacted the HbA_1c_ among DLs.

The number of subjects included in this study did also allow analyzing the impact of the frequency of downloading on glycemic control and therapy success. Our study should be considered as a preliminary study, and based on the results, further information about number of data obtained during the day and downloaded, structured testing, use of additional technologies, and motivation of the subjects might influence the results.

Consistent to the findings that BG downloading improved glycemic control and therapy success in the overall study population and in the subgroup treated with CSII, more frequent downloading resulted in a similar effect, even if the results (due to small number of subjects) were not significant. This prospective cohort study adds evidence that downloading BG data from a BG data management system, which stands for availability and use of BG data, has positive effects on glycemic control and diabetes therapy success.

Finally, we would like to hypothesize that BG data management systems might be effective in reducing the number of ambulatory care visits. It has been estimated that approximately 50% of subjects with T1DM and T2DM make 4 or more visits in 1 year, and in approximately one-third of all visits, no change of therapy is suggested or new drugs are added to titrate therapy [21].

### Future Work

However, even if the observation in this study is encouraging, additional data from larger randomized controlled studies are needed to better identify the clinical setting and the patients who can benefit from the change of care delivered by new technology, including different software available.

In chronic diseases in general, and especially in T1DM, which often occurs at a young age, the need for close collaboration between patients and HCPs, constant checks, and therapeutic adjustments often have a major impact on the quality of life. Technology cannot fully replace personal interaction between patients and physicians but can help to find new ways of delivering care and contribute to therapy success and daily diabetes management.

### Conclusions

In conclusion, BG data management system, which allows collecting, analyzing, downloading, and sharing BG data, offers the opportunity to improve communication between patients and HCPs and connects patients with all stakeholders needed or wanted. The finding of the study supports the importance of BG data download for good glycemic control and diabetes therapy success, as downloading stands for availability and use of BG information. Other functionalities resulting from a connected BG meter to smartphone apps, Web portals, and the cloud also remotely analyzed BG might help in addition to take better therapeutic decisions, potentially decrease the number of office-based visits, and adopt the way diabetes care is delivered. Larger, controlled clinical studies are needed to fully endorse reported findings and support the use of new technologies further.

## Abbreviations

BG: blood glucose
CSII: continuous subcutaneous insulin injection
DCCT: Diabetes Chronic Complications Trial
DL: Downloader
DMS: diabetes management system
FBG: fasting blood glucose
HCPs: health care providers
NDL: No-downloader
SMBG: self-monitoring blood glucose
T1DM: type 1 diabetes mellitus
T2DM: type 2 diabetes mellitus
UKPDS: United Kingdom Prospective Diabetes Study
